# Effect of Flaxseed Meals and Extracts on Lipid Stability in a Stored Meat Product

**DOI:** 10.1007/s11746-014-2438-x

**Published:** 2014-03-01

**Authors:** Katarzyna Waszkowiak, Magdalena Rudzińska

**Affiliations:** Faculty of Food Science and Nutrition, Poznan University of Life Sciences, ul Wojska Polskiego 31, 60-624 Poznan, Poland

**Keywords:** Oxidative stability, Lipid oxidation, Cholesterol oxidation product, Flaxseed extract, Flaxseed defatted meal, Flaxseed variety

## Abstract

Flaxseeds have been recently in focus due to the antioxidant capacity of some of their compounds. However, there is a lack of easily accessible information concerning their activity against lipid oxidation in food systems. Therefore, the aim of the study was to determine the effect of defatted meals (DFM) and the aqueous extracts (AFE) obtained from brown and golden flaxseeds on lipid oxidation in pork meatballs. Fatty acid composition, peroxide value (PV), thiobarbituric acid reactive substances (TBARS) and cholesterol content were monitored during 6 months of freezer storage. Cholesterol oxidation products were identified and quantified. Both DFM and AFE limited fatty acid and cholesterol oxidation during meatball storage. Their antioxidant effect depended on flax variety (brown or golden) and preparation type (DFM or AFE). Lower level of PV and TBARS, compared with the ones with AFE, were noted in meatballs with DFM. Both DFM and AFE, from the brown seed variety, protect the lipids against oxidation to a higher extent. During the storage, a cholesterol degradation was observed. AFE (particularly from the brown variety) limited changes in cholesterol content. Moreover, they stabilized fatty acid composition of stored meatballs. However, DFM efficiently inhibited cholesterol oxidation.

## Introduction

Flax (*Linum usitatissimum* L.) is an important oilseed crop and its seeds are a valuable source of many bioactive compounds [1]. Flaxseed is one of the richest sources of α-linolenic acid, an important source of high-quality protein and soluble fiber [2]. Moreover, the seed contains phenolic compounds [3] such as lignans, phenolic acids (*p*-coumaric, ferulic, *p*-hydroxybenzoic, caffeic, and sinapic acids) and their glucosides, as well as flavonoids (herbacetin and campherol diglucoside). Among the phenolic compounds, flax lignans are in focus because of their estrogenic/antiestrogenic and antioxidant activity [4]. Therefore, flaxseeds and their products are used as a component of functional food [5]. There are numerous examples of flaxseed or oil application in production of bakery goods and some their utilization in meat products. Flax oil was applied to improve fatty acid composition of processed meats [6, 7]. However, oxidative stability of lipids was limited in the products. Bilek and Turhan [8] introduced ground flaxseeds into burgers and they reported that, the higher the addition, the worse the sensory quality of the products (particularly taste). The problem could be solved by using flaxseed extracts that have neutral smell and taste instead of ground flaxseeds. An aqueous extraction of flaxseed meal could improve sensory quality and decrease anti-nutrient (cyanogenic glucoside) content [9]. On the other hand, the extraction process may also affect plant material activity by removing some compounds and changing the concentration of others. Some studies described a synergetic, as well as antagonistic interaction, among plant extract components [10] that may alter their action in food systems when compared to the raw material.

The aim of the study was to determine the effect of defatted flaxseed meals and flaxseed aqueous extracts on lipid oxidation in pork meatballs during storage. In order to analyze the impact of flaxseed composition on the effect, brown and golden varieties were selected as research materials.

## Materials and Methods

### Defatted Flaxseed Meal and Aqueous Extract

Defatted meals and aqueous extracts were prepared from the seeds of three Polish high-α-linolenate flax varieties (IHAR, Poland): brown seeds of the Szafir variety and golden seeds of the Oliwin and Jantarol varieties, as described below. For comparison purposes, a rosemary extract (Naturex, France) was applied in the study.

#### Defatted Meal Preparation

Flaxseeds were ground using a Retch ZM 200 mill (Retch, Germany; 1 mm sieve). Ground seeds were defatted by double cold extraction with petroleum ether according to Kabirullah and Wills [11]. The defatted meal was ground again applying Cyclotec 1039 Sample Mill (Foos Tecator, Sweden) and stored at 4 °C until further use. Partly defatted meals contained 32–35 % of protein, 11–15 % of fat and 9–10.5 % of moisture. Meal with the highest protein and the lowest fat content was obtained from seeds of the Szafir variety.

#### Aqueous Extract Preparation

Defatted flaxseed meal was extracted with water (meal to water ratio of 1:15) [12] under constant stirring with a magnetic stirrer at ambient temperature for 1 h. The precipitate was discarded from the extract after centrifugation (25 min, 1,500×*g*; Centrifuge 5702R, Eppendorf, Germany). The supernatant was collected and freeze-dried (Alpha 1–4 LSC Freeze dryer, Christ, Germany). The extraction process was repeated several times to obtain enough material for the studies to follow. The extracts were pooled and mixed. The average composition of the aqueous extracts were as follows: 34–46 % of protein (the highest was in the case of the Szafir variety) and 6 % of moisture; trace amounts of fat were also detected.

### Meat Ball Production and Storage

Pork (best end of neck) was minced (mesh size of 3 mm); one batch of pork was prepared for all experiments. The minced pork (70 % of meat batter) was mixed with other ingredients such as water (15.8 %), breadcrumbs (8 %), eggs (5 %), salt (1 %) and pepper (0.2 %) [13]. The meat batter was divided into eight portions; one was a control sample, while to the others the following additives (% of meat batter) were blended in: rosemary extract (0.02 %)—RE, defatted flaxseed meals (3 %)—DFM-S, DFM-O, DFM-J and aqueous flaxseed extracts (1.5 %)—AFE-S, AFE-O, AFE-J (the acronyms for preparations obtained from Szafir, Oliwin and Jantarol varieties, respectively). The DFM and AFE concentrations were selected on the basis of the aqueous extraction output (about 8–10 g of AFE was obtained from 20 g of DFM) and by the results of sensory evaluation (DFM and AFE at the selected concentrations did not affect sensory quality of meatballs; unpublished data). Rosemary extract addition was selected according to producer recommendation (by carnosic acid and carnosol content).

After thorough mixing of the additives (approx. 10 min) using a homogenizer, meatballs were formed. The meatballs had a similar weight (50 g) and geometric shape (spherical) to maintain uniform cooking conditions. Meatballs were steamed for 20 min at 100 °C using a convection oven (CCC series, Rational, Germany) and freezer stored (−18 °C) in polyethylene bags for 180 days. During storage, lipid oxidation was periodically assessed by determination of peroxide and thiobarbituric acid reactive substances (TBARS) value. Moreover, fatty acids composition, as well as cholesterol content and cholesterol oxidation products, were monitored.

### Chemical Composition

The chemical composition of the meat balls was determined, that is the content of moisture according to the ISO standard [14], protein (the Kjeldahl method using a Kjeltec-2200 System, Tecator, Sweden) [15] and fat (the extraction-weight method using a Soxtec-HT6 System, Tecator, Sweden) [16].

### Lipid Extraction

Lipids from meat balls were extracted according to the Folch procedure [17] applying a chloroform:methanol solvent system (2:1).

### Determination of Lipid Oxidation

The assessment of the peroxide value (PV) was conducted according to the ISO standard [18] in extracted lipid fraction. The results were expressed as mequiv O_2_ kg^−1^. In turn, 2-TBARS values were determined using the Tarladgis distillation method [19] modified by Pikul et al. [20] and results were expressed as mg malondialdehyde (MDA) kg^−1^.

### Determination of Fatty Acids Composition

The fatty acid composition was estimated by gas chromatography according to AOCS Official Method Ce 1 h-05 [21]. Fatty acid methyl esters were separated using a Hewlett-Packard 5890 II gas chromatograph equipped with a Supelcowax 10 capillary column (30 m × 0.20 mm × 0.20 μm; Supelco, Bellefonte, PA, USA) and an FID detector under programmed temperature conditions: from 60 °C, at a rate of 12 °C min^−1^ to 200 °C and held for 25 min. The temperature of the injection port and detector was held at 240 °C. Identification of separated FAME was performed by comparison of the retention data of separated compounds in the analyzed samples with those obtained for a standard solution.

### Determination of Cholesterol Content

Cholesterol content was determined by AOCS Official Method Ch 6-91 [22]. Briefly, lipids (50 mg) were saponified with 1 M KOH in methanol for 18 h at room temperature, then unsaponifiables were extracted with hexane/methyl *tert*-butyl ether (Sigma-Aldrich, St. Louis, MO, USA) (1:1, v/v). After silylation by Sylon BTZ (Supelco, Bellefonte, PA, USA) cholesterol was separated using gas chromatograph HP 6890 equipped with DB-35MS capillary column (25 m × 0.20 mm; 0.33 μm; J&W Scientific, Folsom, CA). Column temperature was held at 100 °C for 5 min, then programmed to 250 °C at 25 °C min^−1^, held for 1 min, then further programmed to 290 °C at 3 °C min^−1^ and held for 20 min. The detector temperature was set at 300 °C. Hydrogen was used as the carrier gas at a flow rate of 1.5 mL min^−1^. An internal standard, 5α-cholestane (Sigma-Aldrich) was used for sterol quantification.

### Determination of Cholesterol Oxidation Products (COP)

Cholesterol oxides were quantified and identified according to Przygoński et al. [23], as described below.

#### Transesterification

First, 10 mg of the internal standard (19-hydroxycholesterol; Steraloids Inc., Newport, RI, USA) and 2 mL of sodium methoxide (Merck KGaA, Darmstadt, Germany) (10 % in methanol) were mixed with 0.20 g of the lipid sample. The mixture was left for 1 h followed by extraction of the oxysterol fraction with chloroform and then rinsing with water. After separation of the chloroform layer, the extract was evaporated under nitrogen to dryness and dissolved in 250 μL of chloroform.

#### SPE Fractionation

The SEP-PAK NH2 cartridges (Waters Corp., Milford, MA USA) were conditioned with hexane and oxysterol extract was loaded onto the SEP-PAK NH2 column. The column was sequentially eluted with 10 mL of hexane, 5 mL of hexane–MTBE (5:1; v/v), 5 mL of hexane–MTBE (3:1; v/v) and finally with 7 mL of acetone.

#### Derivatization of Oxysterols

To the dried fraction, 100 μL of anhydrous pyridine (Sigma-Aldrich) and 100 μL of BSTFA + 1 % of TMCS (Sigma-Aldrich) were added. After 4 h derivatization at room temperature the sample was ready for GC analysis.

#### GC analysis

Oxidized sterol derivatives were analyzed on a Hewlett-Packard 6890 gas chromatograph equipped with a DB-5 column (30 m × 0.25 mm × 0.25 μm; J&W, Folsom, CA). Samples were injected in a splitless mode and the column temperature programmed as follows: an initial temperature of 160 °C was held for 1 min, then programmed at 40 °C min^−1^ to 270 °C and held for 1 min; further programmed at 4 °C min^−1^ to 280 °C, final temperature was held for 25 min. A hydrogen carrier gas flow of 1 mL min^−1^ was used. Oxysterols were identified using retention data for previously verified compounds by mass spectrometry utilizing our library and published data [24]. Samples from autonomous series were analyzed in triplicate.

### Statistical Analysis

Statistical analyses were conducted using Statistica (version 9.0, StatSoft). Data were expressed as means ± standard deviations (SD) of independent measurements for three samples (*n* = 3). The effect of treatment (addition one of DFM or AFE, RE and control without the addition, *L* = 8) or storage time (days, *L* = 4) was analysed separately. Analysis of variance (ANOVA) for a CRD (completely randomized design) experiment was carried out, and then Tukey’s test at a significance level of *p* ≤ 0.05 was applied to compare the means.

## Results and Discussion

The chemical composition of the meatballs (per 100 g) was approximately as follows: 63.9 ± 0.5 g of moisture, 16.7 ± 0.3 g of protein and 10.3 ± 0.5 g of fat. No significant effect (*p* < 0.05) of flax preparations (DFM and AFE) on the chemical compositions was reported (data not presented).

In the study, lipid oxidation was monitored by changes in PV and TBARS. The results showed no effect of the additions on PV after steaming (1-day storage; Table 1). Higher PV values, than the ones with DFM, were observed for the meatballs with AFE. However, a statistically significant difference was found only between samples with DFM-J and AFE-J.

**Table 1:** Changes in PV [mequiv O_2_ kg^−1^] during freezer (−18 °C) storage of pork meatballs

Time (days)	Additives							
	Control	RE (0.02 %^B^)	DFM (3 %)			AFE (1.5 %)		
			DFM-J	DFM-O	DFM-S	AFE-J	AFE-O	AFE-S
1	0.95 ± 0.06^ab, A^	1.07 ± 0.26^ab^	0.72 ± 0.13^a^	0.62 ± 0.15^a^	0.79 ± 0.27^ab^	1.37 ± 0.02^b^	0.80 ± 0.09^ab^	1.13 ± 0.04^ab^
60	2.60 ± 0.05^e^	1.61 ± 0.04^c^	2.01 ± 0.04^d^	1.38 ± 0.04^b^	0.73 ± 0.04^a^	2.74 ± 0.06^e^	2.18 ± 0.05^d^	1.56 ± 0.06^bc^
120	2.88 ± 0.01^f^	2.06 ± 0.04^d^	2.06 ± 0.01^d^	1.47 ± 0.04^b^	0.81 ± 0.00^a^	2.46 ± 0.03^e^	1.88 ± 0.04^c^	1.45 ± 0.01^b^
180	3.18 ± 0.01^bc^	2.70 ± 0.13^abc^	2.44 ± 0.02^ab^	1.82 ± 0.19^a^	2.04 ± 0.08^a^	5.75 ± 0.60^e^	4.14 ± 0.07^d^	3.43 ± 0.10^cd^

*RE* rosemary extract, *DFM-J*, *DFM-O*, *DFM-S* defatted flaxseed meal from Jantarol, Oliwin and Szafir var., respectively, *AFE-J*, *AFE-O*, *AFE-S* aqueous flaxseed extract from Jantarol, Oliwin and Szafir var., respectively

^A^Mean (*n* = 3) ± SD; means with different letters in the same line are significantly different (one-way ANOVA and Tukey’s test, *p* < 0.05)

^B^% of meat batter

Monitoring of lipid oxidation in steamed meatballs during freezer storage showed a significant effect of storage time, as well as addition, on the changes in PV (*p* < 0.01). The rapid increase of PV was observed for most samples (with the exception of the sample with DFM-S from the brown variety) after a 60-day storage. After a 180-day storage, the increase in the PV again was detected but only for samples with AFE.

The addition of DFM showed a higher inhibition effect against primary oxidation products than in the case of AFE and it was independent of flax variety. Dynamics of changes in the PV of the sample with AFE was at least twice as high for the one with DFM when both were obtained from the same flax variety. Among the samples with DFM, the lowest dynamics of changes in PV was found for the meatballs with brown seed meal (DFM-S). PV recorded for the samples were significantly lower in comparison to the control and at least similar to the one with RE during the entire storage time. In contrast, the PV of the meatballs with AFE of golden seeds (AFE-J and AFE-O) were observed to be significantly higher than the control after 180-day storage.

Monitoring of changes in secondary oxidation products was conducted by determination of the TBARS content (Table 2). The results also demonstrated a significant effect of storage time and addition on the changes (*p* < 0.01). After steaming (1-day storage), lower TBARS contents were observed in the samples with the applied additions compared with the control. A similar trend was found during the entire storage time. At the end of the 180-day storage, the lowest TBARS values were recorded in all samples with DFM, irrespective of varieties, and the highest one in samples with AFE-J. Taking into consideration an inhibition effect of flaxseed preparations against formation of secondary oxidation products during the whole storage, the best solution was to add DFM of the brown seed Szafir var. and the worst to utilize AFE of the golden Jantarol var.

**Table 2:** Changes in TBARS content [mg MDA kg^−1^] during freezer (−18 °C) storage of pork meatballs

Time (days)	Additives							
	Control	RE (0.02 %^B^)	DFM (3 %)			AFE (1.5 %)		
			DEF-J	DEF-O	DEF-S	AFE-J	AFE-O	AFE-S
1	1.01 ± 0.01^f, A^	0.73 ± 0.01^c^	0.82 ± 0.01^d^	0.54 ± 0.01^a^	0.68 ± 0.01^b^	0.84 ± 0.01^d^	0.98 ± 0.01^e^	0.97 ± 0.01^e^
60	1.09 ± 0.02^g^	0.43 ± 0.01^a^	0.66 ± 0.01^b^	1.05 ± 0.01^f^	0.70 ± 0.01^c^	0.64 ± 0.01^b^	1.00 ± 0.01^e^	0.74 ± 0.01^d^
120	1.34 ± 0.01^f^	0.54 ± 0.01^a^	0.78 ± 0.01^d^	0.62 ± 0.01^b^	0.55 ± 0.01^a^	0.83 ± 0.01^e^	0.73 ± 0.01^c^	0.75 ± 0.02^cd^
180	1.01 ± 0.00^d^	0.64 ± 0.01^a^	0.54 ± 0.01^a^	0.54 ± 0.01^a^	0.55 ± 0.01^a^	1.17 ± 0.03^e^	0.55 ± 0.02^a^	0.86 ± 0.01^c^

Acronyms as in Table 1

^A^Mean (*n* = 3) ± SD; means with different letters in the same line are significantly different (one-way ANOVA and Tukey’s test, *p* < 0.05)

^B^% of meat batter

**Fig. 1:**
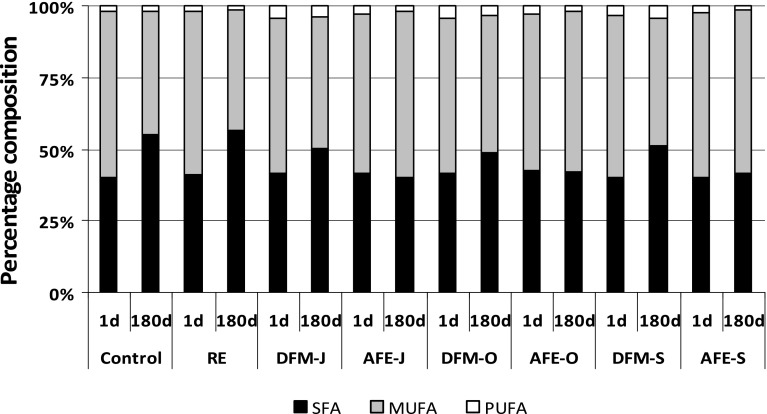
Changes in fatty acids composition (% of total fatty acids) during freezer storage (−18 °C) of pork meatballs. Acronyms of samples as in Table 1; *SFA* saturated fatty acids, *MUFA* monounsaturated fatty acids, *PUFA* polyunsaturated fatty acids; *1d* 1 day of storage, *180d* 180 days of storage. The results are presented as means (*n* = 3) ± SD

The changes in fatty acid composition of meatballs are presented in Fig. 1. Distribution of polyunsaturated (PUFA), monounsaturated (MUFA) and saturated fatty acids (SFA) in the samples with RE and AFE, as well as in the control, was similar after steaming (1 day); it was 2 % of PUFA, 57 % of MUFA and 41 % of SFA. Fatty acid composition of the samples with DFM was characterized by higher level of PUFA (about 4 % of total fatty acids) and lower level of MUFA (54–56 %) and SFA (40–42 %), depending on the flaxseed variety.

After 180 days of storage at −18 °C (Fig. 1; 180d), the contribution of PUFA in the control was unchanged, but MUFA decreased (43.2 %) and SFA increased (54.8 %). A similar change in fatty acid composition was observed in the case of the sample with RE (1.7 % of PUFA, 41.8 % of MUFA and 56.5 % of SFA). Changes in fatty acid composition of meatballs with flaxseed preparations depended on their type (AFE vs DFM). After 180 days of storage, the fatty acid composition of the meatballs with AFE was almost unchanged (1.7–1.8 % of PUFA, 56–58 % of MUFA and 40–42 % of SFA). In the stored samples with DFM, however, the contribution MUFA decreased to 44–48 % and SFA increased to 49–51 %, depending on the flaxseed variety. The lowest changes in fatty acid composition were observed in meatballs with the addition of DFM-S and AFE-S (both from the Szafir var.). It should be mentioned that oil extracted from Szafir seeds [25] are characterized by a lower level of PUFA (67.6 %) and a higher level of MUFA (22.8 %) than those from Oliwin (75.1 and 16.9 %, respectively) and Jantarol var. (71.9 and 20.1 %, respectively), but the contribution of SFA was similar in oils from all the varieties (8–9 %).

The decrease in MUFA during long term storage of the meatballs was probably connected with their oxidation. The classical mechanism for lipid autoxidation is via free-radical attack and unsaturated fatty acids are the initial substrates for the reactions [26]. During freezer storage, the oxidation processes were slowed down but not stopped (Table 1). Some lipid-soluble radicals may even be more stable at lower temperatures and thereby propagate oxidation [27].

**Fig. 2:**
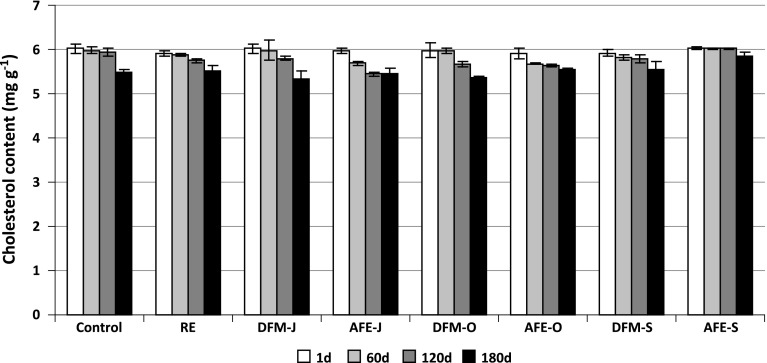
Changes in cholesterol contents (mg g^−1^) during freezer storage (−18 °C) of pork meatballs. Acronyms of samples as in Table 1; *1d* 1 day of storage, *60d* 60 days of storage, *120d* 120 days of storage, *180d* 180 days of storage. The results are presented as means (*n* = 3) ± SD

The cholesterol contents in meatballs with the applied additions and control sample before and after storage are shown in Fig. 2. Phytosterols derived from flaxseeds were detected in the meatballs with DFM, but their amount was very low and was not calculated in the study. The cholesterol level was high because of the ingredients in meatball formulation (eggs and pork fat). After steaming (1 day), the cholesterol content ranged from 5.0 to 6.0 mg g^−1^. During freezer storage of meatballs, cholesterol degradation occurred. After 180 days of storage, the cholesterol content in the control decreased to about 9 % (0.54 mg g^−1^), but about 6.6 % (0.39 mg g^−1^) in the sample with RE. The highest degradation of cholesterol was observed in the meatballs with DFM obtained from golden seeds (DFM-J and DFM-O), that is 11 % (0.68 mg g^−1^) and 10 % (0.61 mg g^−1^), respectively. The lowest degradation of cholesterol was detected in the samples with AFE-S and DFM-S (brown seeds), that is about 2.7 % (0.16 mg g^−1^) and 6.2 % (0.37 mg g^−1^), respectively.

**Fig. 3:**
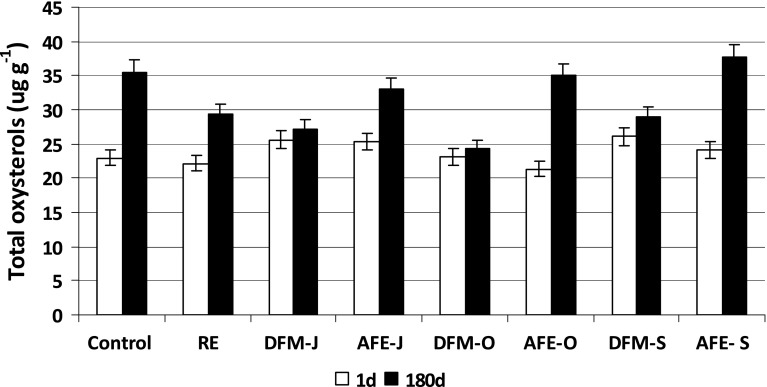
Changes in total oxysterol content (μg g^−1^) during freezer storage (−18 °C) of pork meatballs. Acronyms of samples as in Table 1; *1d* 1 day of storage, *180d* 180 days of storage. The results are presented as means (*n* = 3) ± SD

Cholesterol oxidation products (COP) were determined in the samples after steaming and 180 days of freezer storage. Their total content in all steamed samples ranged from 21 to 26 μg of COP in 1 g of lipids (Fig. 3). After 180 days of freezer storage (180d), the total COP increased by 55 % (12.6 μg g^−1^) in the control sample and by 33 % (7.3 μg g^−1^) in the sample with RE. The highest increase of total oxysterols was found in the meatballs with AFEs and amounted to 30, 64, and 57 % in the samples with AFE-J, AFE-O and AFE-S, respectively. The oxysterol content in the meatballs with DFM, however, did not change significantly during 180 days of storage. The content of total COP increased only by 6 % in the samples with DFM-O and DFM-J (1.3 and 1.6 μg g^−1^, respectively), and about 11 % in the meatballs with DFM-S (2.8 μg g^−1^). Having stoichiometrically converted the amount of oxysterols into cholesterol, it was found that about 1 % of cholesterol was oxidized into these derivatives in the samples with DFM, and 1–9 % in the ones with AFE.

**Fig. 4:**
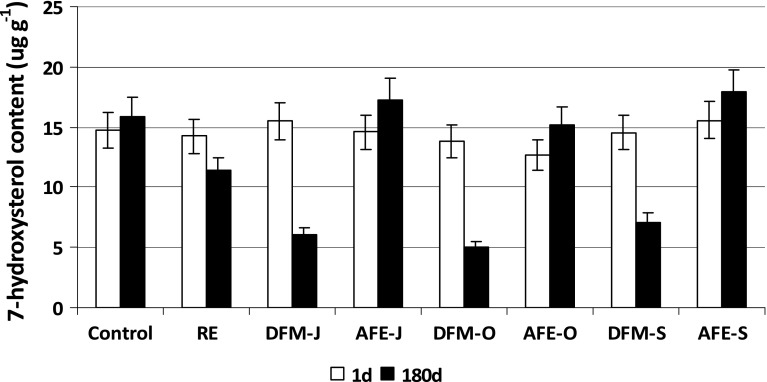
Changes in total 7-hydroxycholesterol content (μg g^−1^) during freezer storage (−18 °C) of pork meatballs. Acronyms of samples as in Table 1; *1d* 1 day of storage, *180d* 180 days of storage. The results are presented as means (*n* = 3) ± SD

The qualitative and quantitative changes in the oxysterol composition were observed during meatball freezer storage. Epimers of 7-hydroxycholesterols (7α- and 7β-OHC) were the principal group of COP in steamed meatballs and their amount ranged 13–16 μg g^−1^ depending on the addition (Fig. 4) and it confirms the results of a previous study [28]. After 180 days of storage (180d), there were no significant changes in 7-OHC content in the control sample, as well as the ones with RE and AFE, but it decreased significantly in the meatballs with DFM. The contributions of both epimers ranged 56–65 % of total COP before storage; after 180 days of storage, it depended on the addition. In the control sample 69 % of 7-OHC was detected and 51 % of 7-OHC in the meatball with RE; in the samples with AFE it ranged 71–74 % and the lowest content (22–27 %) was found in the samples with DFM. It was reported previously [29] that first formed free radicals were located in position C_7_, but during heating and storage they could decompose and give rise to the corresponding hydroxyl derivatives. The shift in hydroxy derivatives formation is related to the accumulation of sterol oxidation products, which are radical acceptors, and affect oxidation [30]. Previous studies also reported that sterol oxidation products were unstable and could interact with one another forming higher molecular weight compounds [31, 32]. Moreover, the formation of the other COP by bimolecular reaction mechanisms was suggested [33].

**Fig. 5:**
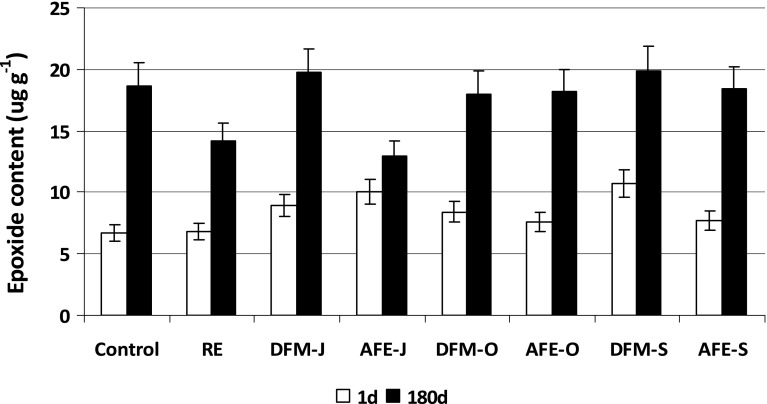
Changes in total epoxycholesterols content (μg g^−1^) during freezer storage (–18 °C) of pork meatballs. Acronyms of samples as in Table 1; *1d* 1 day of storage, *180d* 180 days of storage. The results are presented as means (*n* = 3) ± SD

The content of epoxy derivatives ranged 7–10 μg g^−1^ in all steamed samples and increased during storage time (Fig. 5). The epoxycholesterol contents were similar in the stored control and all the samples with DFM as well as AFE-O and AFE-S. The lowest increases of epoxy derivatives were detected in the meatballs with AFE-J and RE. The percentage contribution of the fraction in total oxysterols was about 30–40 % in steamed meatballs (1 day), and 51–85 % after 180 days of storage. Lercker and Rodriguez-Estrada [29] demonstrated that epoxy derivatives were formed by interaction of a hydroperoxyl radical with cholesterol and the phenomenon was similar to the mechanism observed during monounsaturated fatty acid oxidation.

Triol and 7-ketocholesterol were the minor fraction among oxysterols before and after meatball storage. The highest level of triol was detected in the meatballs with AEF-O before storage (0.4 μg g^−1^) and the one with RE after 180-day storage (1.3 μg g^−1^). The highest 7-ketocholesterol content was detected in the control at the beginning of storage (1.6 μg g^−1^); after 180-day storage, the highest level (2.6 μg g^−1^) was in the sample with RE.

Results of the study have shown that both defatted flax meals and their aqueous extracts obtained from seeds of the selected Polish flax cultivars (Szafir, Oliwin, Jantarol) limit lipid and cholesterol oxidation during freezer storage of steamed meatballs. The earlier results [34] concerning antioxidant capacities and composition of the extracts showed that they exhibited antiradical (DPPH and ABBTS assays) and ferric reducing activities (FRAP assay). Some flaxseed compounds are suspected of being involved in the antioxidant action, i.e. phenolics (lignans and phenolic acids) [3] as well as proteins. Secoisolariciresinol diglucoside (SDG) is known as the main flaxseed lignan. The studies of Prasad [35] and Kitts et al. [36] demonstrated that SDG exhibited radical scavenging ability. The high relationship was found between phenolic content and antioxidant capacity of flax extracts [37, 38]. Moreover, Udenigwe et al. [39] reported some antioxidant activities of flaxseed proteins.

It was found that defatted flax meals more efficiently inhibited oxidation in comparison with aqueous extracts from the same flaxseed variety. The differences in the protective effect between defatted flaxseed meal and aqueous extract obtained from the meal seems to result from differences in their composition. Flaxseed lignans are not extracted with oil [40], therefore, they remain in DFM after defatting. It was found [41] that defatted meals had higher antioxidant activities and a higher antioxidant compound content than their respective seeds and it was explained by changes in the antioxidant concentration after oil extraction.

Previous studies [37, 42] showed that aqueous extracts from defatted flaxseed meals also behaved as antioxidants in a β-carotene/linoleic acid system and the antioxidant activities were affected by their composition (high mucilage in comparison to high protein extracts). However, Waszkowiak et al. [34] found that the extraction method (ethanolic vs. aqueous extraction) greatly affected the phenolic contents of the obtained extracts; the aqueous extraction was less effective at concentrating phenolic compounds. The lower phenolic concentration in aqueous flaxseed extracts seems the most likely explanation of their lower antioxidant efficiency against lipid oxidation when compared to defatted flaxseed meals which was observed in our study.

The study showed differences in the efficiency of inhibition against lipid oxidation among flaxseed preparations obtained from the selected cultivars, i.e., both DFM-S and AFE-S from brown seed of Szafir var. were superior to the ones obtained from golden seeds of Jantarol and Oliwin var. It was probably connected with the differences in chemical composition of the seeds. Lignan content in flaxseeds was found to be cultivar dependent [40]. Results of the earlier study mentioned above [34] showed that among extracts obtained from the selected cultivars (Szafir, Oliwin and Jantarol), the highest antioxidant capacity (radical scavenging and ferric reducing activity) was exhibited by the extracts of Szafir var.; it was correlated with the highest phenolic and protein content in the seeds compared to the seeds of both golden varieties (Jantarol and Oliwin).

## Conclusions

Both defatted meals and aqueous extracts from flaxseeds show an antioxidant effect towards lipids during storage of meat products in a freezer. Therefore, they can be utilized in the meat industry to design products fortified with bioactive compounds of flax seed origin. In addition, they can prolong the shelf-life of the products by protecting them against oxidation processes and their negative impact on sensory and nutritional food quality. However, the antioxidant efficiency of the preparations obtained from flax seeds depends on material composition (flax variety) and preparation procedure that affect active compound concentration. Further research should be carried on to explain this.
